# Deinstitutionalization in Georgia-why it is so slow

**DOI:** 10.1192/j.eurpsy.2024.651

**Published:** 2024-08-27

**Authors:** N. Zavradashvili, G. Matiashvili

**Affiliations:** ^1^School of health sciences, The University of Georgia, Tbilisi, Georgia

## Abstract

**Introduction:**

Mental health reform represents one of the most transformative changes in the field of healthcare, as it not only changes the forms of services but also the nature of services offered. While many countries have successfully implemented such reforms, others, including Georgia, have struggled with a protracted and inconsistent process. Despite decades of advocacy by professionals for deinstitutionalization and the development of community-based services, psychiatric hospital treatment continues to dominate in Georgia.

**Objectives:**

The purpose of the review is to explore the concept of deinstitutionalization within the mental health landscape and assess its status in the context of Georgia. It aims to study the lessons learned from successful deinstitutionalization and illuminate achievements and challenges surrounding deinstitutionalization in Georgia’s reality.

**Methods:**

A qualitative analysis including desk review, in-depth interviews and focus group discussions was conducted. Proceeding from the research objectives we analyzed the existing legislation, strategic documents and clinical practices concerning individuals with mental disorders; Interviews were also conducted with key informants on the shortcomings and problems in deinstitutionalization practices

**Results:**

The review findings reveal, that despite recent progress such as the development of community mobile teams and increased funding allocated for community services within mental health budget, several issues persist: there is no agreement among stakeholders on how to restructure existing hospital beds and financial provisions remain unresolved. The field of mental health in Georgia suffers from a lack of human resources. Attracting new personnel, ensuring regional distribution, and enhancing qualifications are necessary components of deinstitutionalization that require the involvement of all stakeholders, coordinated and time-planned action. The current mental healthcare system in Georgia is characterized by a lack of coordination and collaboration among its various components. Establishing patient care pathways with clear referral criteria is crucial for improving the efficiency of mental health services. This research highlights that successful deinstitutionalization requires additional funds, time, and trained people. Institutions should have a long-term (3-5) year development plan, detailing the source of funding, activities to be implemented, and expected outcomes. In the absence of such a plan, progress remains sporadic, intermittent, uncoordinated, and less effective.

**Conclusions:**

In conclusion, the protracted nature of deinstitutionalization in Georgia requires a multifaceted approach involving various stakeholders. By addressing identified challenges and promoting coordination among mental health components, Georgia can guide a more effective course toward a community-based, patient-centered mental healthcare system.

**Disclosure of Interest:**

None Declared

